# Supplementary material for: Plasticity of body axis polarity in Hydra regeneration under constraints
Source: Sci Rep. 2022 Aug 3;12:13368. doi: 10.1038/s41598-022-17411-9 (PMC9349251; doi:10.1038/s41598-022-17411-9)
Supplement: Supplementary file 1 — Supplementary Information. [file 41598_2022_17411_MOESM1_ESM.pdf]

Supplementary Information

Plasticity of body axis polarity in *Hydra* regeneration under constraints

Anton Livshits [*], Liora Garion[*], Yonit Maroudas-Sacks, Lital Shani-Zerbib, Kinneret Keren[#], Erez Braun [#]

Supplementary movies

Movie 1. H2H doublet that regenerated into an animal with a head in the middle. Time-lapse movie of a H2H doublet that underwent polarity reversal, regenerating into an animal with a head in the middle and two feet at the edges. Combined epifluorescence (Left; green- AlexaFluor 647-conjugated 10kD dextran, magenta- Texas Red-conjugated 3kD dextran) and bright-field (Right) images are shown. The elapsed time from excision is displayed (hrs:min), and the scale bar is 200 µm.

Movie 2. H2H doublet that regenerated a head on its edge. Time-lapse movie of a H2H doublet that underwent polarity reversal, regenerating into an animal with a normal morphology, with a head that developed from an originally foot-facing side of one of the excised rings. Combined epifluorescence (Left; green- AlexaFluor 647-conjugated 10kD dextran, magenta- Texas Red-conjugated 3kD dextran) and bright-field (Right) images are shown. The elapsed time from excision is displayed (hrs:min), and the scale bar is 200 µm.

Movie 3. H2F doublet in the oriented configuration that regenerated along its original polarity. Time-lapse movie of an oriented H2F doublet that regenerated into a normal animal along the original body axis orientation. Combined epifluorescence (Left; green- AlexaFluor 647-conjugated 10kD dextran, magenta- Texas Red-conjugated 3kD dextran) and bright-field (Right) images are shown. The elapsed time from excision is displayed (hrs:min), and the scale bar is 200 µm.

Movie 4. H2F doublet in the anti-oriented configuration that reversed polarity. Time-lapse movie of an anti-oriented H2F doublet that regenerated into an animal whose body axis is oriented in the opposite direction to its original polarity, with a head forming on the original foot-facing edge. Combined epifluorescence (Left; green- AlexaFluor 647-conjugated 10kD dextran, magenta- Texas Red-conjugated 3kD dextran) and bright-field (Right) images are shown. The elapsed time from excision is displayed (hrs:min), and the scale bar is 200 µm.

Movie 5. F2F doublet that regenerated into a normal morphology with a single head. Time-lapse movie of a F2F ring doublet that regenerated into an animal with a single head on the U side. Combined epifluorescence (Left; green- Texas Red-conjugated 3kD dextran, magenta- AlexaFluor 647-conjugated 10kD dextran) and bright-field (Right) images are shown. The elapsed time from excision is displayed (hrs:min), and the scale bar is 200 µm.

Movie 6. F2F doublet that regenerated two heads. Time-lapse movie of a F2F ring doublet that regenerated into an animal with two heads on both sides. Combined epifluorescence (Left; green- Texas Red-conjugated 3kD dextran, magenta- AlexaFluor 647-conjugated 10kD dextran) and bright-field (Right) images are shown. The elapsed time from excision is displayed (hrs:min), and the scale bar is 200 µm.

Movie 7. Actin organization in a single regenerating ring. Time-lapse, spinning-disk confocal movie depicting the actin organization in a regenerating ring. The oral side of the ring was labeled with a fluorescent tissue label (by locally uncaging Abberior CAGE 552) to mark the original polarity of the tissue, which is preserved in the regeneration process. Right: projected lifeact-GFP signal showing the organization of the ectodermal actin fibers. Left: overlay of the lifeact-GFP signal (green) with the fluorescent tissue label (blue). The elapsed time from excision is displayed in hours (hrs:min), and the scale bar is 100 µm.

Movie 8. Actin organization in H2H doublet that underwent polarity reversal. Time-lapse, spinning-disk confocal movie depicting the actin organization in a H2H doublet that underwent polarity reversal and regenerated into an animal with a head on an originally foot-facing side of the labeled ring. The aster-like defect at the labeled edge is clearly visible from the beginning of the movie, and coincides with the site of head formation in the regenerated animal. Right: projected lifeact-GFP signal showing the organization of the ectodermal actin fibers in the ring doublet. Left: overlay of the lifeact-GFP signal (green) with the fluorescent tissue label marking one of the rings (blue; Texas Red-conjugated 3kD dextran). The elapsed time from excision is displayed in hours (hrs:min), and the scale bar is 100 µm.

Movie 9. Actin organization in H2H doublet that regenerated a head in the middle. Time-lapse, spinning-disk confocal movie of a H2H doublet that regenerated into an animal with a head in the middle and two feet at both edges. A +1 defect (surrounded by two -1/2 defects) forms *de novo* at the tip of a protrusion that appears at the middle of the regenerating doublet and

develops into a middle head. Right: images of the projected lifeact-GFP signal showing the organization of the ectodermal actin fibers in the ring doublet. Left: overlay of the lifeact-GFP signal (green) with the fluorescent tissue label marking one of the rings (blue; Texas Red-conjugated 3kD dextran). The elapsed time from excision is displayed in hours (hrs:min), and the scale bar is 100 μm.

Supplemental Figures

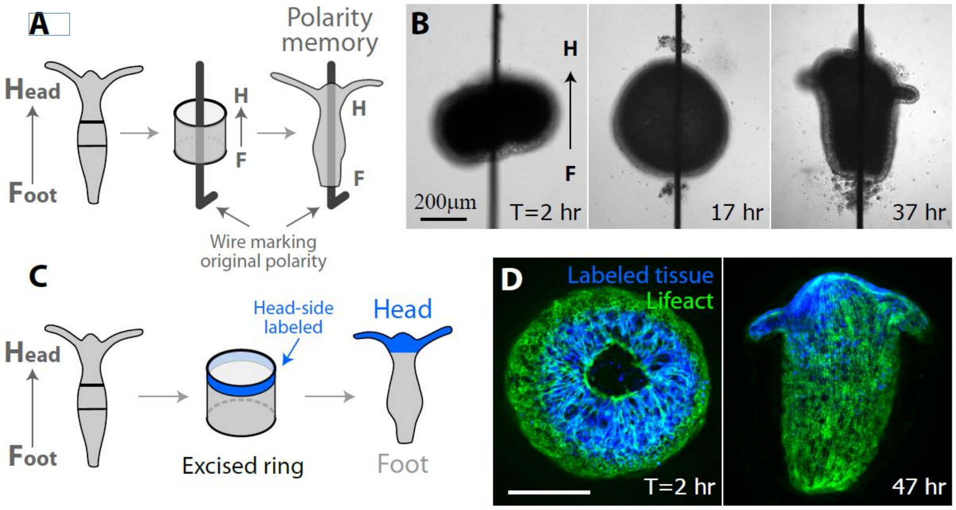

Figure S1. Memory of polarity in regenerating single tissue rings. (A,B) Memory of polarity in single tissue rings regenerating on a wire**.** (A) Schematic illustration of the regeneration of an excised tissue ring on a wire [37]. A ring is excised from the gastric region of a mature *Hydra*. The ring is threaded on a wire that is bent at one end to mark the original polarity of the tissue. The majority of excised rings observed were able to regenerate on the wire (61 out of 92 samples regenerated, 12 escaped from the wire, 8 disintegrated, and 11 did not regenerate). 60 out of the 61 regenerated samples had a normal morphology that maintained the original polarity of the excised ring (1/61 samples regenerated into an abnormal morphology). (B) Images from a time lapse movie of an excised ring that regenerated on a wire. The original polarity of the excised ring is maintained in the regenerated animal. (C,D) Memory of polarity in a regenerating tissue ring that is labeled at its oral side. (C) Schematic illustration of the regeneration of a tissue ring excised from the gastric region of a mature animal. The excised ring regenerates into an animal with a new head at the labeled region and a new foot at the other end, thus maintaining its original polarity. (D) Spinning-disk confocal images from a time-lapse movie of an excised ring that is labeled at its oral end (Movie 7). The labeling is done by locally uncaging an electroporated caged-dye (Abberior CAGE 552; see Methods) [38]. Images show an overlay of the projected lifeact-GFP signal (green) and the fluorescent tissue label (blue) that marks the tissue originating from the oral edge of the tissue ring shortly after excision (left) and in the regenerated animal (right).

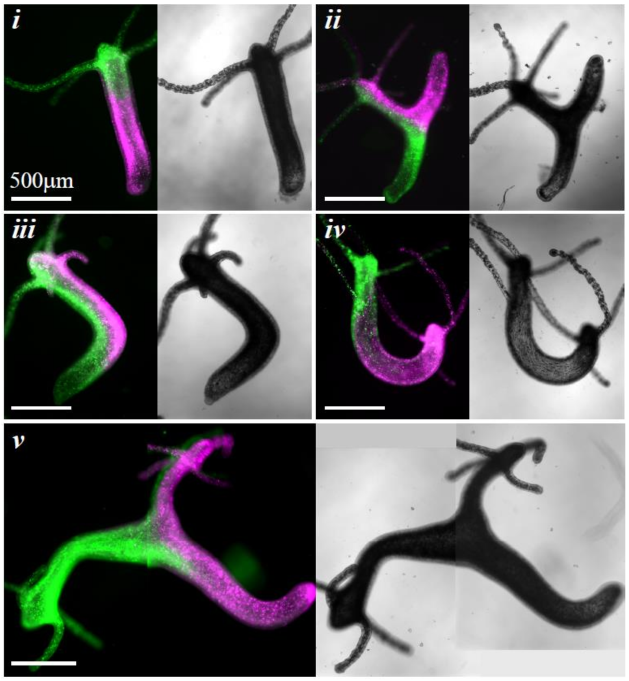

Figure S2. Different types of morphological outcomes in regenerating ring doublets. Combined epifluorescence (Left; green- AlexaFluor 647-conjugated 10kD dextran, magenta- Texas Red-conjugated 3kD dextran) and bright-field (Right) images of different outcome morphologies observed in the regeneration of ring doublets in the various configurations: (i) Normal (ii) Head in the middle with two feet (iii) Head originating from the middle of the doublet with normal morphology (iv) Two heads at opposite sides and (v) Heads in the middle and on the side.

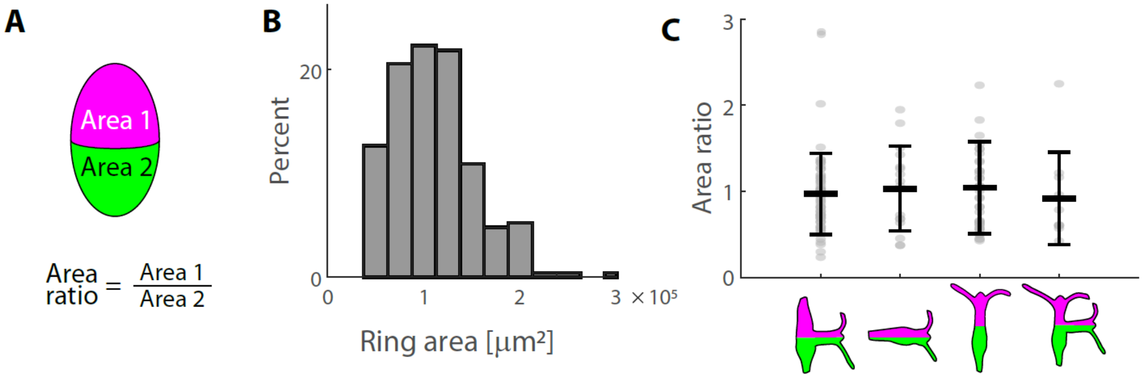

Figure S3. The relative sizes of fused tissue rings in H2H doublets: The size of the tissue originating from the two rings was characterized by the projected area within the fused spheroid (N=114 H2H doublets; see Methods). (A) Schematic illustration showing the projected areas, and the area ratio which provides a measure of the relative sizes of the two rings. (B) Bar plot showing the size distribution of projected ring areas (N=228 rings). (C) A graph depicting the area ratio of regenerating H2H ring doublets as a function of the possible morphological outcomes. For each type of outcome, the mean area ratio and standard deviation are shown (black), as well as a scatter plot of the area ratio values for each ring doublet (gray dots).

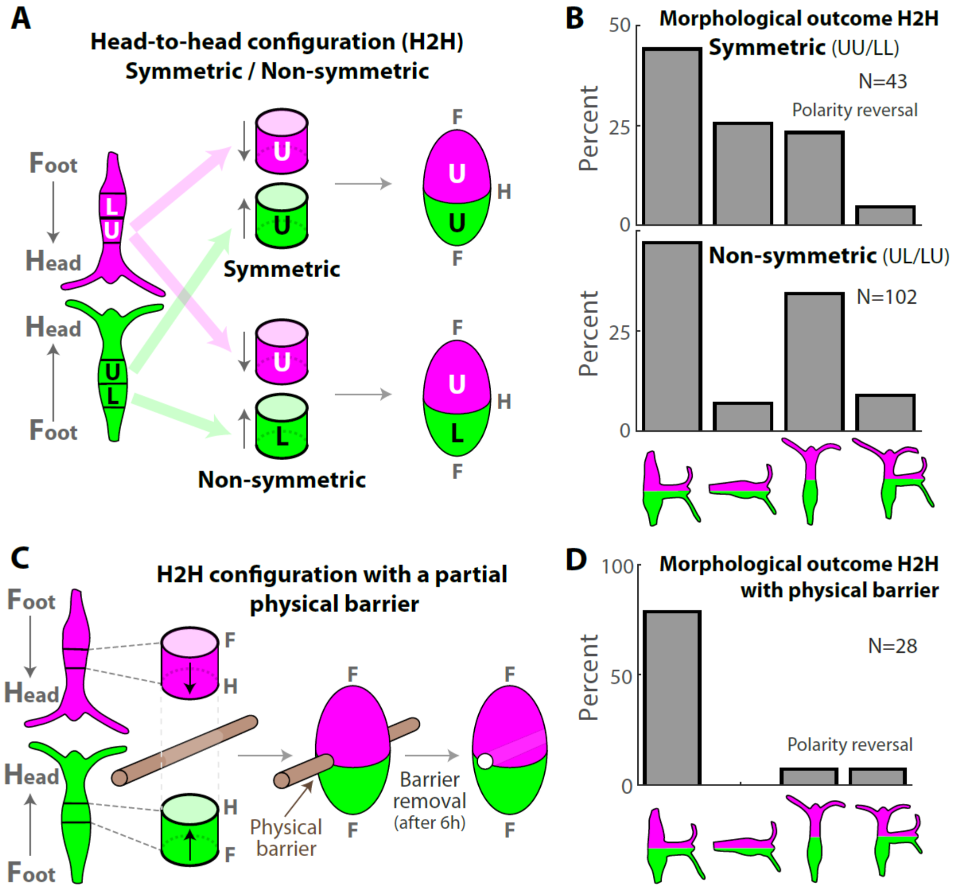

Figure S4. Detailed analysis of the regeneration of H2H doublets. (A) Schematic illustration of the regeneration experiments with fused ring doublets in the H2H configuration. Rings are excised from above (U) or below (L) the approximated midpoint of the two parent animals that are differentially labeled (green/magenta). The rings are fused so that their originally head-facing sides adhere to each other. Samples are made from two rings taken from the same position along the body axis of the parent animals (top; symmetric UU or LL) or from different positions (bottom; non-symmetric UL or LU). (B) Bar plot depicting the outcome morphologies of fused H2H ring doublets generated in a symmetric (UU or LL; top) or non-symmetric (UL or LU; bottom) manner. (C) Schematic illustration of the formation of a H2H doublet with a physical barrier (a 75 µm-diameter wire) placed at the adhesion site. The barrier is removed after ~6 hours and the regeneration proceeds as in (A). (D) Bar plot depicting the outcome morphologies of H2H doublets formed with this physical barrier. The probability for polarity reversal with head formation at an originally foot-facing side of one of the excised rings (right bars) is reduced by the presence of the barrier (compare to (B)).

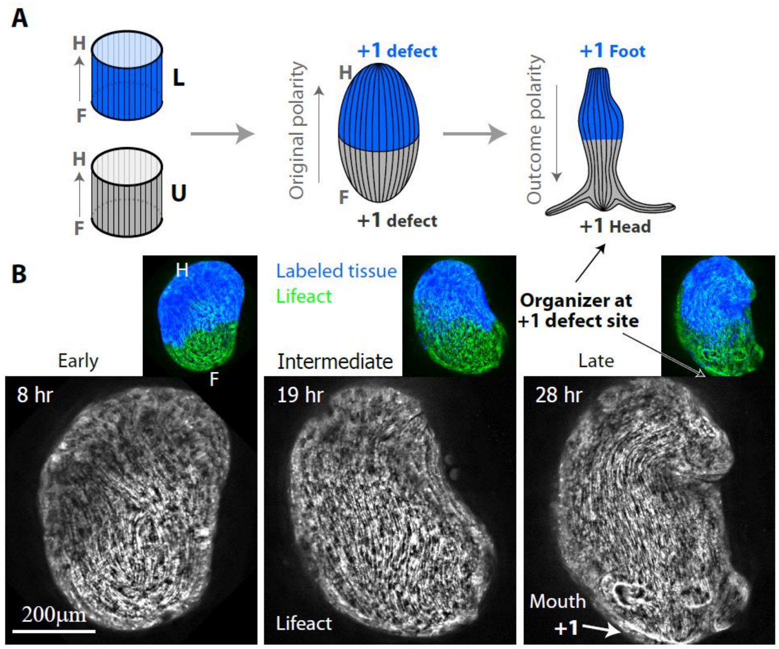

Figure S5. Actin fiber organization in a regenerating H2F ring doublet that undergoes polarity reversal. (A) Schematic illustration of the ectodermal actin fiber organization during regeneration of an anti-oriented H2F ring doublet that undergoes polarity reversal. Left: The actin fibers in the excised rings are arranged in parallel arrays, along the direction of the body axis of their parent animal. The two rings are positioned in an anti-oriented H2F configuration, and one of the rings is marked with a fluorescent tissue label (blue). Middle: Following fusion, the actin fibers from the two rings can join to form continuous fibers that span the length of the fused ring doublet. Two aster-like defects form at the top and bottom ends of the ring doublet. Right: In the case of polarity reversal, the fused ring doublet regenerates into an animal that has a normal morphology, with a new head forming at the aster-like (+1) defect site at the bottom (originally foot-facing) edge of the doublet, and a foot forming at the other (originally head-facing) end. (B) Spinning-disk confocal images from a time-lapse movie of an anti-oriented H2F doublet that underwent polarity reversal. Images are shown at an early (left), intermediate (middle) and late (right) time points during the regeneration process. The projected lifeact-GFP signal (see Methods) shows the organization of the ectodermal actin fibers. The +1 defect site at the bottom edge of the sealed doublet coincides with the formation site of the new organizer at the mouth of the regenerated animal. Insets: Overlay depicting the lifeact-GFP signal (green) together with the fluorescent tissue label that marks one of the fused rings (blue; Texas Red-conjugated 3kD Dextran).
